# Cross‐Cultural Influences on the Association Between Rumination and Psychopathology: A Systematic Review

**DOI:** 10.1002/jclp.70135

**Published:** 2026-03-31

**Authors:** James Haoxiang Li, Julian Madsen, Joshua Wong, Belinda Liddell, Laura Jobson

**Affiliations:** ^1^ Turner Institute for Brain and Mental Health and School of Psychological Sciences Monash University Melbourne Victoria Australia; ^2^ School of Psychology University of New South Wales Sydney New South Wales Australia; ^3^ School of Psychological Sciences University of Newcastle Callaghan Newcastle Australia

## Abstract

Rumination is a transdiagnostic process associated with psychopathology. While culture shapes cognitive and emotion processing, cultural influences on rumination remain unclear. Therefore, this systematic review aimed to examine cultural differences in the association between rumination and psychopathology. To address this aim, we conducted a literature search (May 2024), which identified 24 eligible studies. We conducted an exploratory meta‐analysis examining whether cultural group moderated the association between rumination and psychopathology. First, we found cultural differences in the association between rumination and psychopathology under certain conditions. Second, we identified three culture‐specific mechanisms that may shape the relationships between rumination and psychopathology: (1) social support was less eroded by rumination in Japanese versus German participants and correlated with weaker rumination‐wellbeing associations; (2) self‐doubt attributions mediated the association between rumination and depression symptoms in European Americans but not Asians; and (3) happiness levels moderated the relationship between rumination and depression symptoms in European Americans but not Asian Americans. Third, given the limited studies available, meta‐analyses could only be conducted comparing Western and Asian samples for depression symptoms. This exploratory analysis highlighted cultural group did not significantly moderate the relationship between rumination and depression. Finally, the review highlighted the scarcity of sufficient studies to draw definitive conclusions about the role of culture in rumination and psychopathology and the need for research focusing on diverse cultural groups, clinical samples and disorder‐specific measures of rumination. Advancing this research is crucial for informing the integration of culture into theories of rumination and enhancing cultural tailoring of interventions.

Rumination is a maladaptive cognitive process with adverse effects on mental health. It is characterized by repetitively dwelling on one's negative feelings and their causes and consequences (Nolen‐Hoeksema 1991). Extensive research demonstrates rumination is consistently associated with anxiety, depression and PTSD (Cano‐López et al. 2022; Moulds et al. 2020; Rickerby et al. 2022). Non‐clinical research has also found rumination to be associated with lower subjective well‐being (Harrington & Loffreido 2011). Thus, rumination has been established as a transdiagnostic maintenance process and frequently targeted in psychological interventions (McLaughlin and Nolen‐Hoeksema 2011; Mendoza et al. 2022).

Multiple theoretical accounts outline how rumination contributes to poorer mental health. While it is beyond the scope of the article to review all models, here we highlight several of the most prominent models to demonstrate the central role of rumination in the development, maintenance and treatment of psychopathology. One of the earliest accounts of rumination and psychopathology emerged from the Response Styles Theory (Nolen‐Hoeksema 1991), which outlines mechanisms through which rumination may contribute to negative mood, and thus depression. Rumination exacerbates distress by (1) amplifying negative cognitions through fixation on negative memories and self‐criticism (Nolen‐Hoeksema 1991), (2) hindering effective problem‐solving by interfering with one's attention and concentration (Lyubomirsky and Nolen‐Hoeksema 1995), and (3) eroding social support by creating aversive social experiences for others (Nolen‐Hoeksema and Davis 1999). Following the seminal Response Styles Theory, theoretical accounts of the role of rumination in maintaining other psychological disorders rapidly emerged. The Contrast Avoidance Model (Kim and Newman 2022; Newman and Llera 2011) posits that rumination may function as an emotion regulation strategy in both depression and anxiety by sustaining negative affect in order to avoid sharp increases in negative affect when faced with distressing situations. However, in the long‐term this ultimately perpetuates distress and emotion regulation difficulties (for empirical evidence see Jamil and Llera 2021). In anxiety and PTSD, rumination is thought to reinforce conditioned fear responses and interfere with habituation (Erikson et al. 2019; Foa et al. 2009). Specific to PTSD, rumination may contribute to the fragmented and biased nature of trauma memories, where abstract themes of fear are easily accessed, while discrete contextual details are not properly consolidated (Williams et al. 2007). Rumination is thought to exacerbate these memory biases through dwelling on abstract themes, consequently impeding adaptive processing of the trauma required for recovery and maintaining maladaptive appraisals of the trauma (Williams et al. 2007). Notably, while rumination involves repetitive negative thoughts across disorders, it likely manifests differently across disorders. In depression, it centres on causes and consequences of current distress; in anxiety, it blends with future‐oriented worry; and in PTSD, it may focus more traumatic memories (Ehring 2021).

Research has further differentiated between subtypes of rumination. For instance, Treynor et al. (2003) proposed a distinction between brooding and reflection. Brooding is characterized by a passive preoccupation with negative aspects of oneself or negative interpretations of one's circumstances (Treynor et al. 2003). In contrast, reflection is characterized by a purposeful engagement in cognitive problem‐solving, motivated by self‐curiosity (Treynor et al. 2003). Studies have shown inconsistencies between these two types of rumination and their associations with psychopathology; while brooding is consistently associated with worse outcomes, there is no clear link found for reflection (Cox et al. 2012; Joormann et al. 2006; Treynor et al. 2003). Another distinction has been made by Andrews and Thomson (2009), who posited that causal analysis and problem‐solving analysis are distinct processes that occur during rumination. Causal analysis is characterized by repetitively attempting to understand the causes of one's negative emotions or negative life events, often resulting in self‐blame. In contrast, problem‐solving analysis is characterized by analytical attempts to identify options to improve a situation or to prevent similar problems in the future (Andrews and Thomson 2009). While there are mixed findings relating to the maladaptive effects of causal analysis (Bartoskova et al. 2018; Maslej et al. 2020; Sevcikova et al. 2020), problem‐solving analysis is consistently negatively associated with depressive symptoms (Bartoskova et al. 2018; Sevcikova et al. 2020). Thus, rumination is likely a multidimensional construct, and the form and content of rumination may influence its adaptiveness.

While there have been significant theoretical and empirical advances guiding our transdiagnostic understanding of rumination, a critical limitation remains existing theories are based largely on research conducted in Western cultural contexts and overlook the role of culture. This is a key issue for multiple reasons. First, while clinical guidelines worldwide emphasize the importance of culturally competent practice (e.g., American Psychological Association 2017; National Institute for Health and Care Excellence 2018; Australian Health Practitioner Regulation Agency 2024) and rumination remains a key treatment target, there is currently little scientific understanding in this area to guide such practices, presenting a key challenge to clinicians. Moreover, research shows that interventions have poorer outcomes when cultural factors are ignored but improve significantly with cultural tailoring (Huey and Tilley 2018). Given the prevalence and impact of psychopathology globally, and the tendency to ruminate being universal, there is an urgent need to investigate how culture may influence the relationship between rumination and psychopathology.

This need becomes particularly clear when examining fundamental cultural differences that may alter the function of rumination in the context of psychopathology. Theoretical work has highlighted key dimensions by which cultural groups systematically vary, which have important implications for rumination's function across cultures. These dimensions include cultural values, cognitive style, and view of emotions. Some cultures (predominantly Western) tend to hold an *independent* self‐construal, whereby the self is conceived as unique, independent, and detached from the social context (Markus and Kitayama 2010). In contrast, other cultures (e.g., Asian) tend to adopt an *interdependent* self‐construal, where one's interconnectedness and harmony with others is emphasized to a greater degree (Markus and Kitayama 2010). Cultures can also vary based on cognitive style. Some cultural groups (predominantly Western), tend to endorse *analytic thinking styles*, characterized by distinguishing focal points from their contexts, and ascribing causality to events based on formal logic (Nisbett et al. 2010). In contrast, other cultures (predominantly Eastern) tend to adopt a *holistic* thinking style, characterized by paying attention to relationships between objects and their contexts (Nisbett et al. 2010).

By synthesizing the theories of self‐construal and cognitive style, de Vaus et al. (2018) conceptualized pathways through which these cultural factors may influence rumination. Cultural groups that are likely to have independent self‐construal and analytic thinking styles may be more likely to ruminate from a self‐immersed perspective and fixate on the content of their own negative emotions. These individuals may also be inclined to understand negative emotions as caused by their internal qualities, rather than external circumstances, thus evoking greater distress (de Vaus et al. 2018). These patterns are contrasted with other cultural groups that are likely to have interdependent self‐construal and holistic thinking styles. Rumination in these cultures may be a more self‐distanced process, whereby individuals view their experiences with a broader focus on social and contextual factors. This, in turn may facilitate adaptively gaining insight and closure over persistent negative thought patterns, rather than re‐experiencing of negative emotion (see de Vaus et al. 2018 for full review).

Cultural understandings of emotion may also influence rumination. Western cultures tend to highly value positive emotions and perceive negative emotions as undesirable (Zhang and Cross 2011). When combined with a tendency to believe in events remaining constant, a characteristic of analytic thinking styles (Nisbett et al. 2010), this may result in getting ‘stuck’ in negative emotions and seeing them as a permanent obstacle or threat to happiness (de Vaus et al. 2018). In contrast, those from Asian cultures tend to endorse a more balanced rating of both positive and negative emotions (Zhang and Cross 2011). In conjunction with a greater tendency to believe that the world, and thus emotions are constantly changing, a characteristic of holistic thinking styles, this may leave members of these cultures to be less likely ‘stuck’ in their negative emotions, while leaving more capacity for flexible reflection (de Vaus et al. 2018; Nisbett et al. 2010).

There is accumulating empirical evidence for these cultural understandings of rumination in the context of psychopathology (Potthoff et al. 2016; Schunk et al. 2021; Xu and Tsai 2023). Recent studies have found that culture moderates the association between rumination and psychopathology, such that rumination is less associated with negative outcomes in Asian when compared to Western groups (Jobson et al. 2022; Choi and Miyamoto 2022; Toussaint et al. 2023). However, not all studies have found that culture moderates these associations (Li et al. 2022; Nagulendran and Jobson 2020). Critically, despite clear theoretical accounts suggesting rumination is associated with psychopathology (Mendoza et al. 2022), and the cross‐cultural theories and research suggesting culture influences rumination (de Vaus et al. 2018; Grossmann and Kross 2010), to date, there is no comprehensive summary of all available evidence relating to cultural influences on rumination in the context of psychopathology. This presents challenges to clinicians, who consequently do not have an evidence‐base to draw upon when considering cultural variations in rumination in clinical interventions, limiting their ability to provide culturally responsive care.

## 1 Aims and Hypotheses

To address these gaps in the literature, this systematic review aimed to synthesize existing evidence to clarify cultural influences on the relationship between rumination and psychopathology, highlighting key themes and trends. Second, we sought to understand what factors may underlie or drive any potential cultural differences (e.g., form/focus of rumination, cultural values etc.). Third, we sought to critically appraise the methodology used in the existing research and provide directions for future cross‐cultural investigations. Finally, we aimed to where possible conduct exploratory meta‐analyses examining whether cultural group moderated the association between rumination and psychopathology outcomes. This final aim was exploratory as we were not certain the number of studies that would meet the inclusion criteria for each psychopathology outcome. Additionally, while we aimed to compare across a diverse range of cultural groups, it became apparent that there were not enough studies to conduct these analyses. Hence, guided by current theories and research on culture and emotion (de Vaus et al. 2018; Grossmann and Kross 2010), which focus on Western and Asian cultural groups, we decided to focus the meta‐analysis on Western versus Asian cultural groups. We hypothesized that greater rumination would be associated with greater psychopathology. Second, we hypothesized that cultural group would moderate this association between rumination and psychopathology, such that rumination would show a stronger association with greater symptomatology (e.g., depression, anxiety) in Western groups when compared to other collectivistic cultural groups (for the meta‐analyses this pertained to Asian cultural groups).

## 2 Methods

### 2.1 Data Source and Search Strategy

We conducted a systematic review utilising the Preferred Reporting Items for Systematic Review and Meta‐Analysis (PRISMA) framework to identify articles that investigated cultural differences in the association between rumination and psychopathology. The research design involved generating a search strategy, study screening, quality review, data extraction and reporting. The systematic review protocol was registered *a priori* with PROSPERO #CRD42022321392, including the aims, search strategy and inclusion/exclusion criteria. A university librarian was consulted while developing the search strategy for each database. We conducted the literature search using PsycINFO, Scopus, PubMed, and MedLine in June 2022, and again in May 2024. A publication date‐range from 1990 to 2024 (inclusive) was applied, and both free text words and MeSH headings were used. Search terms were informed by existing literature, theory, and constructs. See Supporting File 1 for full list of search terms.

Articles were included in the review if they met the following criteria: (1) the article compared the association between rumination and psychopathology (including well‐being) between at least two cultural groups (thus including cross‐national comparisons as well as studies comparing different cultural groups within the same country e.g., European Americans *vs.* Asian Americans), (2) participants were adults (18 years or older), (3) the article was published in English, and (4) the article was an empirical study or dissertation. For the meta‐analysis, additional criteria were applied: (1) studies must compare the association between a Western and Asian cultural group (as noted above, this included studies comparing different countries as well as cultural groups within the same country), (2) sufficient data (e.g., correlation coefficients) are reported to calculate effect sizes for each cultural group and (3), for each psychological disorder (e.g., depression) at least five studies must be available to ensure robust estimates and prevent high risk of bias (Hedges and Vevea 1998). There was no restriction set on the types of studies included; randomized controlled trials, quasi‐experimental and cross‐sectional studies were all included if the inclusion criteria were met. Studies involving both clinical and healthy populations were included.

### 2.2 Screening Process

Screening adhered to the Cochrane Collaboration Guidelines (Lefebvre et al. 2019). Sources from databases were uploaded into Covidence Reference Management Software, with duplicates removed. The authors also hand‐searched key journals and reference lists to identify potentially relevant studies not found through electronic databases. The primary reviewer (HL) undertook screening of titles and abstracts against the inclusion criteria. An additional reviewer (JM) independently screened the results at the title and abstract level. A third reviewer (LJ) was consulted to resolve any discrepancies between the two reviewers when making the final decision. Adhering to Cochrane Collaboration Guidelines, the primary reviewer (HL) carried out the full‐text screening independently to ensure inclusion criteria were met.

### 2.3 Data Extraction and Synthesis

Data extraction was carried out by the primary reviewer (HL) and verified by a secondary reviewer (JM). Extracted data included author(s), study name, design, participant characteristics, measures, cultural modifications (i.e., any cultural changes made to measures or paradigms used) and key findings. No secondary data analysis was undertaken. The number of studies addressing each outcome variable (e.g., life satisfaction, depression, anxiety) were reported to examine the research available for each psychological symptom.

### 2.4 Quality Assessment

A customised quality assessment tool was constructed to evaluate the methodological quality of studies. The assessment was adapted from the National Institutes of Health Quality (NIH) Assessment Tool for Observational Cohort and Cross‐Sectional Studies (National Heart Lung Blood Institute 2018). To assess classifications of cultural group, we added a question: ‘Was an appropriate standard used to measure cultural group?’. Two reviewers (HL, JM) independently conducted the quality review alongside data extraction. Responses were compared and there was a 90% agreement between raters. Disagreements were resolved through discussion.

### 2.5 Exploratory Meta‐Analytic Analyses

We conducted an exploratory meta‐analysis with moderator analyses to examine the influence of cultural group (Western *vs.* Asian) on the association between rumination and each psychopathology outcome variable. Specifically, we sought to examine if effect sizes differed between Western and Asian cultural groups. Studies were included if they measured psychopathology outcomes either as a continuous variable based on self‐reported symptom severity in healthy or dysphoric samples, or as a binary variable when comparing dysphoric participants to healthy control participants. A meta‐analysis was not conducted for outcome variables where there were less than five studies cross‐culturally examining its association with rumination, given a higher risk of bias (Hedges and Vivea 1998).

### 2.6 Calculation of Effect Sizes

Comprehensive Meta‐Analysis Version 4 (CMA; Borenstein et al. 2022) was used to conduct the meta‐analysis. The Pearson correlation coefficient (r) was used as the effect size, as it is easily interpretable (Kallogjeri and Piccirillo 2023). One study reported effect sizes using partial eta squared (Mihailova and Jobson 2020). Therefore, this was converted to Pearson's r using formulas provided by Cohen (1988).

To perform the meta‐analyses, Pearson correlation coefficients were transformed through Fisher's z transformation to correct for the r coefficients' problematic standard error formulation (Lipsey and Wilson 2001). Following the analyses, summary Fisher's z values, and associated 95% confidence intervals were converted back to Pearson correlation coefficients for reporting and interpretation (Borenstein et al. 2009). Using Cohen (1988) conventions, values of 0.10 were considered small, values of 0.30 were considered moderate, and values of 0.50 were considered large. Categorical moderator analyses (cultural group) were performed employing an analogue to analysis of variance, with results interpreted using the random effects model where common within‐study variance was assumed for cultural groups by using a pooled estimate of within‐subgroup variance (Borenstein et al. 2009).

### 2.7 Heterogeneity

As significant between‐study heterogeneity was expected due to variations in cultural groups and outcome measures, a random effects model was selected a‐priori. To assess heterogeneity, Cochran's Q test and the I^2^ index were utilised. The Q statistic examines whether the effect sizes across studies are consistent, with a significant result suggesting variability beyond sampling error, thus rejecting the null hypothesis of homogeneity (Higgins and Thompson 2002; Lipsey and Wilson 2001). The *Q* statistic was also used to assess heterogeneity between different cultural groups when performing the moderator analyses. Due to limited power of the Q test in smaller meta‐analyses and its inability to quantify heterogeneity, we additionally calculated the *I* ^*2*^ statistic (Higgins and Thompson 2002). The *I* ^*2*^ statistic quantifies the degree of variance between study results reflecting variance in true effects rather than sampling error (Higgins and Thompson 2002; Higgins et al. 2003). *I* ^*2*^ values range from 0% to 100% with larger values indicating higher heterogeneity; heterogeneity around 50% is considered moderate and heterogeneity around 75% is considered high (Higgins et al. 2003).

### 2.8 Sensitivity and Measurement and Publication Bias

To address potential measurement and publication bias arising from the underrepresentation of non‐significant findings, we examined funnel plots displaying effect sizes against their standard errors. Funnel plot asymmetry, an indication of missing studies due to publication bias, was assessed using Egger's test (Egger et al. 1997). Orwin's fail‐safe N (Orwin 1983) was also computed to estimate the number of unpublished studies with a mean effect‐size of 0 would be required to reduce the overall effect size to a specified level. This effect size threshold was set to *r* = 0.10 (Orwin 1983).

## 3 Results

### 3.1 Search Results

**Figure 1:**
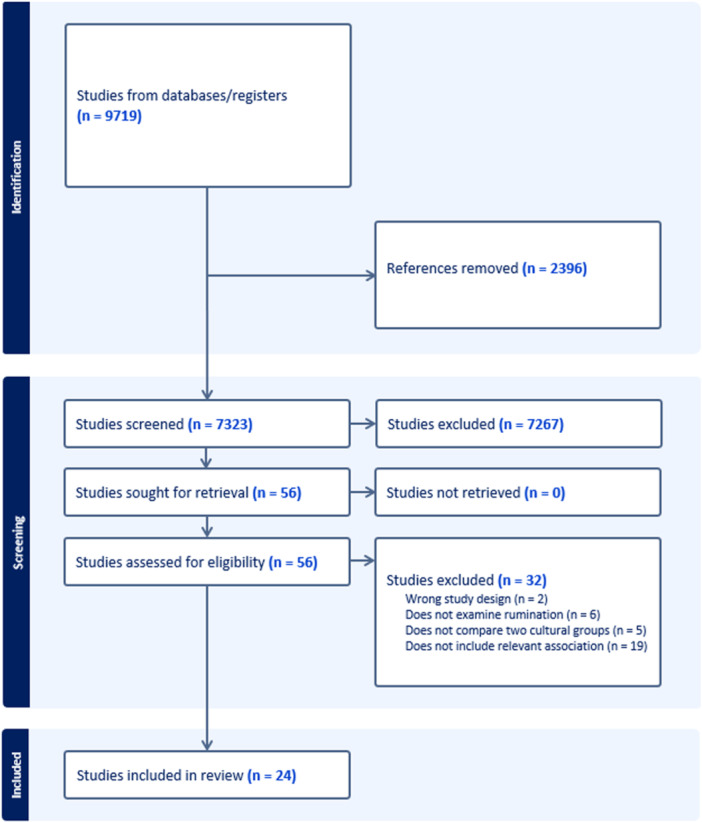
PRISMA flow diagram of study selection.

As outlined in Figure 1, the initial database search identified 9719 references, of which 2396 were duplicates and deleted. Next, 7323 studies were screened against title and abstract, with 7267 studies excluded, and the remaining 56 studies assessed for full‐text eligibility. After full‐text review, 24 studies met the inclusion criteria and were included for data extraction and synthesis. The studies had overall moderate‐high level of research quality indicating a good strength of evidence and trustworthiness (see Supporting File 2).

### 3.2 Descriptive Statistics and Study Characteristics

Table 1 presents a summary of key descriptive characteristics and main findings for each study. All studies but one (*n* = 23, 96%) employed a cross‐sectional design, which employed a quasi‐experimental design (Tsai and Lau 2013). All studies utilized self‐report questionnaires as a measure of both rumination and psychopathology. Half of the studies (*n* = 12, 50%) utilized university student samples. No studies have been conducted in clinical samples or examined participants with a clinical diagnosis.

**Table 1:** Summary of studies included in the review.

Authors	Design and sample	Rumination measure	Well‐being measure(s)	Key findings
Bravo et al. (2019)	Cross‐sectional University students; US (*n* = 924), Argentina (*n* = 403) Spain (*n* = 305)	Ruminative Thought Style Questionnaire (Brinker and Dozois 2009)	**Depression Symptoms:** CESD Scale (Radloff (1977))	In all three groups, problem focused, counterfactual, repetitive and anticipatory thoughts were correlated with depression symptoms.
Chang et al. (2010)	Cross‐sectional 422 Asian American (*n* = 184) and European American (*n* = 238) college students	RRS (Nolen‐Hoeksema and Morrow 1991; Treynor et al. 2003)	**Positive and Negative Affect:** 20‐item Positive and Negative Affect Schedule (Watson et al. (1988)) **Depression Symptoms:** BDI (Beck (1961)). **Anxiety:** BAI (Beck et al. (1988)).	Rumination was less strongly associated with positive affectivity, negative affectivity, depression symptoms and anxious symptoms for Asian Americans compared to European Americans. Rumination shared less variance with negative affect, depressive symptoms and anxious symptoms in Asian Americans than in European Americans.
Cheref et al. (2015)	Cross‐sectional Undergraduate Students; Black American (*n* = 216), Latino American (*n* = 395), Biracial (*n* = 79)	RRS (Nolen‐Hoeksema and Morrow 1991; Treynor et al. 2003)	**Depression Symptoms:** BDI‐II (Beck et al. (1996)) **Hopelessness:** Beck Hopelessness Scale (Beck et al. (1988)) **Suicidal Ideation:** Beck Scale for Suicidal Ideation (Beck et al. (1993))	In all three groups, brooding was associated with depression symptoms and hopelessness. Brooding was associated with suicidal ideation in the Black American and Latino American groups, but not the biracial group. In all three groups, reflection was associated with depression symptoms. Reflection was associated with hopelessness in the Black American and Latino American groups, and suicidal ideation in the Black American and biracial groups.
Choi and Miyamoto (2022)	Cross‐sectional 265 European American (*n* = 142) and East Asian (*n* = 123) undergraduates	RRS‐Short Form Brooding and Reflective subscales (Nolen‐Hoeksema 1991; Treynor et al. 2003)	**Depression Symptoms:** CESD Scale (Radloff (1977)) **Anxiety:** State‐Trait Anxiety Inventory (Spielberger 1983)	Rumination was more strongly associated with depression symptoms in European American participants than East Asian participants. There were no differences between cultural groups in the association between rumination and anxiety.
Eshun (2000)	Cross‐sectional University Students; Caucasian (*n* = 105) African (*n* = 89)	Response Styles Questionnaire ‐ Rumination subscale (Nolen‐Hoeksema and Morrow 1991)	**Suicidal Ideation:** Adult Suicidal Ideation Questionnaire (Reynolds (1991))	Rumination was correlated with, and independently predicted, suicide ideation in both cultural groups.
Grossmann and Kross (2010)	Cross‐sectional University Students: American (*n* = 86), Russian (*n* = 76)	RRS Brooding subscale (Treynor et al. 2003)	**Depression Symptoms:** BDI (Beck et al. (1996))	Russians who identified as self‐reflective showed less depression symptoms than self‐reflective Americans. The relationship between self‐reflection and depression symptoms was stronger in Americans than Russians.
Jobson et al. (2022)	Cross‐sectional 207 European American (*n* = 104) and Asian American (*n* = 103) trauma survivors	Repetitive Thinking Questionnaire (McEvoy et al. 2010) RRS Short Form Brooding subscale (Treynor et al. 2003)	**Depression Symptoms:** HADS (Zigmond and Snaith (1983)) **PTSD Symptoms:** PCL‐5 (Weathers et al. (2013))	Brooding and PTSD symptoms were more strongly correlated in the European American group than the Asian American group. The two groups had similar levels of PTSD symptoms at low levels of rumination, however the European American group had higher levels of PTSD symptoms than the Asian American group at high levels of rumination.
Kwon et al. (2013)	Cross‐sectional University Students American (*n* = 384), Korean (*n* = 380)	RRS Short Form Brooding and Reflection subscales (Treynor et al. 2003)	**Depression Symptoms:** CESD Scale (Radloff (1977))	Association between reflection rumination and depression symptoms stronger in Americans compared to Koreans. Association between brooding rumination and depression symptoms similar in both countries.
Li et al. (2022)	Cross‐sectional Trauma survivors: 253 Australian (*n* = 109) and Malaysian (*n* = 144)	RRS Short Form Brooding subscale (Treynor et al. 2003)	**Depression Symptoms:** HADS Depression subscale (Zigmond and Snaith (1983)) **PTSD Symptoms:** PCL‐5 (Blevins et al. (2015))	Brooding rumination was positively associated with depression symptoms and PTSD symptoms in both the Malaysian and Australian groups separately. No evidence that cultural group moderated associations between brooding rumination and depression/PTSD symptoms.
Mason and Lewis (2017)	Cross‐sectional Female university students: Caucasian (*n* = 100), African‐American (*n* = 84)	Response Styles Questionnaire – Brooding subscale (Treynor et al. 2003)	**Binge Eating:** Report number of days in the past week the participant “rapidly consumed an excessive amount of food with an experience of loss of control”	Rumination was positively associated with likelihood of binge eating for both groups.
Mihailova & Jobson (2020)	Cross‐sectional Adults: European Australian (*n* = 46), East Asian (*n* = 45)	RRS Brooding subscale (Treynor et al. 2003)	**Depression Symptoms:** BDI‐II (Beck et al. (1996))	The East Asian depressed group brooded significantly more than the European Australian depressed group. However, control groups did not differ significantly.
Miranda et al. (2013)	Cross‐sectional University Students; White (*n* = 230), Ethnic minority (*n* = 479)	RRS (Nolen‐Hoeksema et al. 1999)	**Depression Symptoms:** PHQ‐9 (Spitzer (1999))	Brooding and reflection were associated with depression in ethnic minority participants (adjusting for perceived discrimination and ethnic identity). Brooding, but not reflection, was associated with depression symptoms in White participants (adjusting for perceived discrimination and ethnic identity).
Nagulendran and Jobson (2020)	Cross‐sectional Trauma Survivors: East Asian Australian (*n* = 38) Caucasian Australian (*n* = 31)	Habitual rumination: RRS (Nolen‐Hoeksema and Morrow 1991) Trauma‐Specific Rumination: Responses to Intrusions Questionnaire (Clohessy and Ehlers 1999)	**PTSD Symptoms:** Clinician‐Administered PTSD Scale for DSM‐5 (Weathers et al. (2013))	Rumination was associated with PTSD symptoms in the overall sample, and both East Asian Australian and Caucasian Australian groups separately. There was no evidence to suggest habitual rumination differed between cultural groups.
Niu (2013)	Cross‐sectional Adults: Chinese American (*n* = 57), US American (*n* = 29)	RRS (Nolen‐Hoeksema and Morrow 1991) Stress Reactive Rumination Scale (Robinson and Alloy 2003)	**Depression Symptoms:** Geriatric Depression Scale Short Form (Brink et al. (1982)) **Suicidal Ideation:** Geriatric Suicidal Ideation Scale (Heisel and Flett (2006))	RRS Total and Brooding were associated with depression symptoms in non‐Chinese adults but not Chinese adults. RRS Total and Brooding were associated with suicidal ideation in both Chinese and non‐Chinese adults.
Potthoff et al. (2016)	Cross‐sectional Adults from universities or general community in Netherlands *(n* = 301), Hungary (*n* = 235), Spain (*n* = 394), Italy (*n* = 154), Portugal (*n* = 367), Germany (*n* = 102)	Cognitive Emotion Regulation Questionnaire (Garnefski and Kraaij 2007)	**Depression Symptoms:** BDI‐II (Beck et al. (1996)); Patient Health Questionnaire (Kroenke et al. (2001)); Brief Symptom Inventory (Derogatis (1993)) **Anxiety Symptoms:** Symptom Checklist‐90 (Derogatis and Cleary (1977)); State‐Trait Anxiety Inventory (Spielberger 1983); Anxiety‐Sensitivity Index‐3; Brief Symptom Inventory (Derogatis (1993))	Rumination was positively correlated with depression symptoms and anxiety in all country samples except Italy.
Ramos‐cejudo et al. (2017)	Cross‐sectional Adults: Australian (*n* = 637) and Spanish (*n* = 1115)	Anger Rumination Scale (Sukhodolsky et al. 2001)	**Depression Symptoms:** PHQ‐9 (Kroenke et al. (2001)) **Anxiety:** Generalized Anxiety Disorder‐7 (Spitzer et al. (2006))	Angry afterthoughts, thoughts of revenge, angry memories and understanding of causes were all associated with depressive symptoms and anxiety symptoms in both the Australian and Spanish groups. The associations are slightly weaker in the Spanish sample compared to the Australian sample.
Schunk et al. (2021)	Cross‐sectional University Students: German (*n* = 148), Hong Kong Chinese (*n* = 125) Japanese (*n* = 127)	Perseverative Thinking Questionnaire (Ehring et al. 2011)	**Perceived Social Support:** Multidimensional Scale of Perceived Social Support (Zimet et al. (1988) **Life Satisfaction:** Satisfaction with Life Scale (Diener et al. (1985))	Rumination was associated with lower life satisfaction across all cultural groups; culture did not moderate this relationship. Culture moderated the relationship between rumination and social support; rumination was associated with less social support in German and Hong Kong Chinese, but not in Japanese participants. Social support partially mediated the negative relationship between rumination and life satisfaction in German and Hong Kong Chinese participants.
Schunk et al. (2022)	Cross‐sectional 1000 Japanese (*n* = 524) and Austrian/German *n* = 476) university students	Perseverative Thinking Questionnaire (Ehring et al. 2011)	**Depression Symptoms:** CESD Scale (Radloff (1977)) **Subjective Well‐being:** Combination of the Satisfaction with Life Scale (Diener et al. (1985)) and the Scale of Positive and Negative Experience (Diener et al. (2010))	Rumination predicted lower subjective well‐being. Culture moderated this relationship such that the relationship was significantly weaker in the Japanese group compared to the Austrian/German group. Rumination predicted more depression symptoms; however, culture did not moderate this relationship.
Schunk et al. (2023)	Cross‐sectional University Students: German (*n* = 129), Hong Kong Chinese (*n* = 136), Japanese (*n* = 123)	Perseverative Thinking Questionnaire (Ehring et al. 2011)	**Life satisfaction:** Satisfaction with Life Scale (Diener et al. (1985)) **Perceived social support from friends:** Multidimensional Scale of Perceived Social Support (MSPSS; Zimet et al. (1988))	Across cultures, rumination was associated with lower life satisfaction and unrelated to social support by friends.
Taku et al. (2009)	Cross‐sectional US adults (*n* = 224), Japanese undergraduates (*n* = 431)	Rumination Scale (Calhoun et al. 2000)	**Posttraumatic Growth (PTG):** Posttraumatic Growth Inventory (Taku et al. (2009))	Rumination as correlated with PTG. Deliberate rumination soon after the event was a significant predictor of PTG in the Japanese sample but not the US sample, over and above recent rumination. In both samples, intrusive rumination did not predict PTG.
Toussaint et al. (2023)	Cross‐sectional University students; Korea (*n* = 204), US (*n* = 297)	RRS (Treynor et al. 2003)	**Depression Symptoms:** BDI‐II (Beck et al. (1996))	Rumination was associated with depression symptoms in both cultures, but more strongly in US sample than Korean sample.
Tsai et al. (2011)	Cross‐sectional 422 Asian American (*n* = 184) and European American (*n* = 238) college students	RRS (Nolen‐Hoeksema and Morrow 1991; Treynor et al. 2003)	**Depression Symptoms:** BDI (Beck (1961)) **Anxiety:** BAI (Beck et al. (1988))	The interaction between rumination and happiness predicted anxious or depressive symptoms over and above main effects for European Americans, but not Asian Americans.
Tsai and Lau (2013)	Quasi‐experimental 192 Asian American (*n* = 100) and European American (*n* = 92) undergraduate students	12‐item adapted scale: participants rated their engagement in 12 types of ruminative/reflection responses using a 7‐point Likert scale ranging from 1 (Strongly disagree) to 7 (Strongly agree). (Tsai and Lau 2013)	**State affect:** Questionnaire developed for response‐style studies (Morrow and Nolen‐Hoeksema (1990); Wisco and Nolen‐Hoeksema (2009))	Participants in both interpersonal rejection and achievement failure groups experienced more post‐reflection distress than those in the control group. Asian Americans in the IR condition were more distressed than European Americans.
Xu and Tsai (2023)	Cross‐sectional Adults: European American (*n* = 235), Asian Americans (*n* = 198), Latinx Americans (*n* = 168)	RRS (Treynor et al. 2003) Analytical Rumination Questionnaire – casual analysis and problem‐solving subscales (Barbic et al. 2014; Bartoskova et al. 2018)	**Depression Symptoms:** CESD Scale (Radloff (1977)) **Life satisfaction:** Satisfaction with Life Scale (Diener et al. (1985))	Brooding was associated with greater depression symptoms and lower life satisfaction in all ethnoracial groups. Causal analysis was associated with higher levels of depression symptoms among Asian and Latinx Americans but not among European Americans Problem‐solving analysis was associated with lower depression symptoms and greater life satisfaction among Asian and Latinx Americans, but not among European Americans.

*Note:* RRS = Ruminative Responses Scale; CESD = Center for Epidemiological Studies Depression; BDI = Beck Depression Inventory; BAI = Beck Anxiety Inventory; PCL‐5 = PTSD Checklist for DSM‐5; HADS = Hospital Anxiety and Depression; PHQ‐9 = Patient Health Questionnaire‐9. * We classified Russia as “Asian” rather than “Western” due to its shared cultural characteristics with Asian countries that differ markedly from Western individualistic cultures. Prior research (e.g., Realo and Allik 1999; Varnum et al. 2010; Grossmann and Kross 2010) has grouped it with other collectivist/interdependent societies due to being more similar in cultural values and contrast it with Western European/American cultures. This aligns with our goal of testing hypotheses about cultural moderation.

Methods of defining cultural groups differed between studies. Several studies (*n* = 9, 37.5%) defined cultural groups based solely on country of residence (e.g., Australia vs China). Other studies (*n* = 14, 58%) defined cultural groups based on ethnicity (e.g., Asian Australian *vs.* European American). A few studies (*n* = 4, 17%) had inclusion criteria requiring participants to identify as having a particular cultural heritage and having all four grandparents with the same cultural heritage.

In measuring rumination, the Ruminative Responses Scale (RRS) was the most frequently used measure (*n* = 12, 54%). Many studies using the RRS focused only on the brooding subscale (*n* = 5, 21%), with a smaller number of studies using both the brooding and reflection subscales (*n* = 4, 16%), and some studies (*n* = 3, 12.5%) utilizing the RRS total score as an index of rumination. Other tools used to assess rumination included the Response Style Questionnaire (*n* = 4, 17%), the Pervasive Thinking Questionnaire (*n* = 3, 12.5%), Anger Rumination Scale (*n* = 1, 4%), Cognitive Emotion Regulation Questionnaire (*n* = 1, 4%), Rumination Scale (*n* = 1, 4%) and Zimbardo Time Perspective Inventory (*n* = 1, 4%).

In considering psychological symptom outcomes, more than half of the studies (*n* = 15, 62.5%) examined depression symptomatology. Other variables included subjective well‐being (*n* = 5, 20%), anxiety symptoms (*n* = 4, 17%), PTSD symptoms and post‐traumatic growth (PTG) (*n* = 4, 17%), suicidal ideation (*n* = 3, 12.5%), and binge eating (*n* = 1, 4%).

### 3.3 Overview of Empirical Findings

#### 3.3.1 Depression

Fifteen studies compared the association between rumination and depression symptomatology across cultural groups. Nine studies compared Western (e.g., European Americans) and Asian (e.g., Korean, Asian American) groups, with five finding a weaker association in Asian groups (Chang et al. 2010; Choi and Miyamoto 2022; Grossmann and Kross 2010; Toussaint et al. 2023; Xu and Tsai 2023), and four studies finding no cultural influence (Kwon et al. 2013; Li et al. 2022; Schunk et al. 2022; Schunk et al. 2023). Three studies comparing Western groups (i.e., Argentina, Australia, Hungary, Netherlands, Germany, Portugal, Spain, US) consistently reported a moderate to strong association between rumination and depression symptomatology, with no evidence of cultural group moderating the association (Bravo et al. 2019; Potthoff et al. 2016; Ramos‐cejudo et al. 2017).

#### 3.3.2 Anxiety

Four studies explored the associations between rumination and symptoms of anxiety, with two comparing Western and Asian groups. The findings were inconsistent. Chang et al. (2010) found significantly weaker associations between rumination and anxiety symptoms in Asian Americans when compared with European Americans. In contrast, Choi and Miyamoto (2022) did not find a moderating effect of culture (Eastern vs Western). Two studies examining different European groups reported rumination was significantly associated with anxiety symptoms across all European countries included in these studies (Bravo et al. 2019; Potthoff et al. 2016).

#### 3.3.3 Subjective Well‐Being

Five studies explored cultural differences in the association between rumination and subjective well‐being. Four such studies investigated life satisfaction as a component of subjective well‐being. Two of these studies additionally investigated positive and negative affect and followed Hitokoto and Uchida's (2015) approach by combining frequency of positive and negative emotions and life satisfaction into a single subjective well‐being component score to ensure representation of multiple aspects of the construct.

All of the well‐being studies compared participants from Western cultural heritage (i.e., German, European American) with participants of Asian cultural heritage (i.e., Asian American, Hong Kong Chinese, Japanese). Three of these studies found culture influenced this association. Chang et al. (2010) found that the association between rumination and both positive and negative affectivity were significantly weaker for Asian Americans compared to European Americans. Similarly, Schunk et al. (2022) found that culture moderated the association between rumination and well‐being, such that this association was significantly weaker in Japanese participants compared to German participants. Xu and Tsai (2023) found that problem‐solving analysis, a subtype of rumination, was associated with lower depressive symptoms and greater life satisfaction among Asian Americans, but not among European Americans. In contrast, two studies did not find any cultural influence on the association between rumination and life satisfaction (Chang et al. 2010; Schunk et al. 2021). One quasi‐experimental study elicited rumination by instructing participants to recall a memory involving interpersonal rejection or achievement failure (Tsai and Lau 2013). Asian Americans experienced more heightened distress compared to European Americans when purposefully reflecting on a negative interpersonal experience. However, there was not a significant difference when the reflection content was about an achievement failure.

#### 3.3.4 PTSD and PTG

All three studies comparing the associations between rumination and PTSD symptomatology cross‐culturally found increased rumination was associated with PTSD symptoms in Asian and Western trauma survivors. These results were consistent for brooding rumination (Li et al. 2022; Jobson et al. 2022), habitual rumination (Nagulendran and Jobson 2020), and trauma‐related rumination (Jobson et al. 2022). There were mixed findings on cultural moderation. Two studies found that cultural group did not moderate the relationship between rumination and PTSD symptoms (Li et al. 2022; Nagulendran and Jobson 2020). In contrast, Jobson et al. (2022) found that the association between brooding rumination and PTSD symptoms was weaker in an Asian American group compared to a European American group.

One study (Taku et al. 2009) investigated the influence of culture on the association between rumination types and PTG. They found for both Japanese and Americans, recent deliberate rumination predicted the current extent of PTG, highlighting its protective role. However, some cultural differences emerged. For US participants, recent use of deliberate rumination was more important than deliberate rumination immediately post‐trauma in PTG. However, for the Japanese sample, both rumination post‐trauma and recent rumination were positively associated with PTG.

#### 3.3.5 Suicidal Ideation and Binge Eating

The three studies investigating cross cultural differences in the association between rumination and suicidal ideation consistently found brooding rumination to be related to suicidal ideation pan‐culturally. This was found when comparing Chinese with non‐Chinese older adults (Niu 2013), Caucasian Americans with Ghanaians (Eshun 2000) and Blacks and Latinos (Cheref et al. 2015). One study reported rumination being associated with binge eating in both Caucasian and African American women (Mason and Lewis 2017).

### 3.4 Meta‐Analysis Results

**Figure 2:**
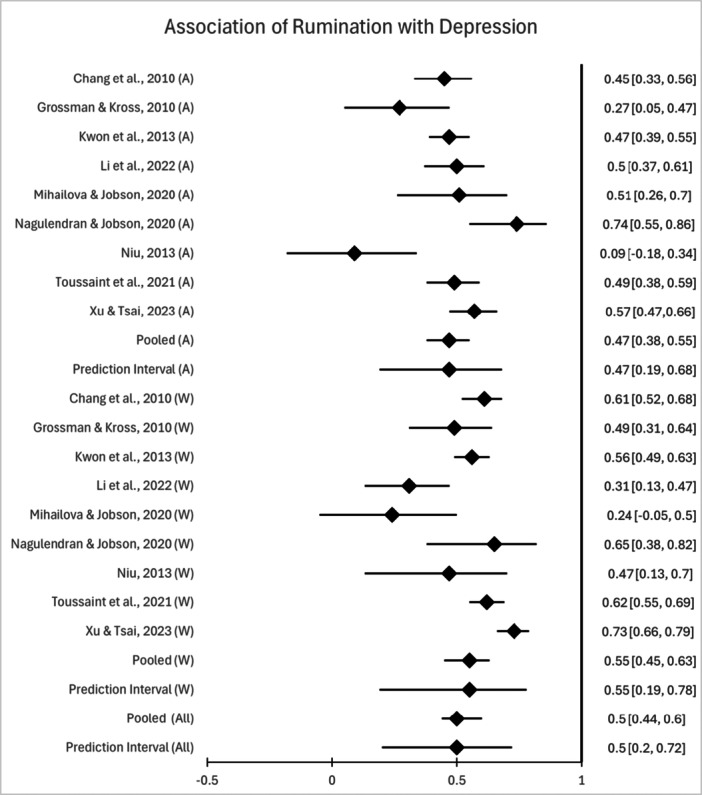
Forest Plot of the association between rumination and depression. Studies are listed on the left‐hand side. Asian samples are marked as (A) and Western samples are marked as (W). The overall correlations (grand effect) are marked as (All).

A meta‐analysis was conducted exclusively for depression symptomatology and not conducted for any other outcome variables, as there were less than five studies cross‐culturally examining their associations with rumination, presenting a higher risk of bias (Hedges and Vivea 1998). A random‐effects model was employed for the meta‐analysis examining the relationship between rumination and depression symptomatology. Figure 2 presents a forest plot of the associations between rumination and depression symptomatology across different cultural groups, with individual study effect sizes and their 95% confidence intervals displayed. As shown in Table 2, a large positive correlation (*r* = 0.50, 95% CI [.44, 0.56]) was found between rumination and depression symptomatology. The consistency of this association across most studies, despite minor cultural differences, suggests rumination is strongly linked to depression symptomatology.

**Table 2:** Random weighted mean effect sizes and heterogeneity statistics associated with rumination by cultural group.

Variable	*k*	*N*	Range of raw *rs*	Mean *r*	95% CI	*Z*‐value	*Q‐*value	*I* ^2^
Overall	18	2,212	0.09–0.74	0.50	[0.44, 0.56]	13.40***	81.07***	79.03
Asian	9	547	0.09–0.74	0.47	[0.38, 0.55]	9.65***	24.46***	67.29
Western	9	431	0.24–0.73	0.55	[0.45, 0.63]	9.38***	40.48***	80.24

*Note:* k = number of studies; *N* = total sample for meta‐analysis; *r* = Pearson *r* correlation; 95% CI = 95% confidence intervals; Q‐value = Q statistic for homogeneity; *I* ^2^ = proportion of total variance attributable to between‐study variance.

* *p* <0.05

** *p* < 0.01

*** *p* < 0.00.

*Heterogeneity analyses*. A significant *Q*‐statistic was observed, indicating that between‐study homogeneity could not be assumed (Cochran 1954). Additionally, *I* ^*2*^ index statistics indicated a large amount of variability (79%) was attributable to between‐study heterogeneity (see Table 2) (Higgins and Thompson 2002; Higgins et al. 2003). This suggests that observed differences in effect sizes across studies are not due to random chance alone but rather reflect systematic differences in how rumination relates to depression symptomatology depending on contextual factors such as study methodology and cultural groups investigated.

*Moderator analyses*. Random effect moderator analyses indicated that cultural group (Western vs. Asian) did not significantly moderate the relationship between rumination and depression symptomatology, *Q*‐between = 1.67, *df* = 1, *p* = 0.20.

*Publication bias*. Visual inspection of the funnel plot did not suggest significant asymmetry of effect sizes. Additionally, non‐significant Egger's tests suggested that significant publication bias was not present b0 = −1.71, *SE* = 1.32, *t* = 1.29, one‐tailed *p* = 0.10. Publication bias was also assessed using Orwin's fail‐safe N tests (Orwin 1983), which indicated that to reduce our overall summary correlations to *r* = 0.10, 90 additional unpublished studies with effect sizes of 0 was needed. This suggests the results are not due to missing studies.

### 3.5 Cultural Differences in Rumination Subtypes

Five studies investigated rumination subtypes and their associations with psychopathology across cultures. Three of these studies compared Western and Asian groups, with contrasting findings. Brooding was consistently associated with depression symptoms in two studies (Kwon et al. 2013; Xu and Tsai 2023). However, Kwon et al. (2013) found the association between reflection and depression symptoms was weaker in Koreans than Americans in Kwon et al. (2013), while Xu and Tsai (2023) found the association between problem‐solving analysis and both depression symptoms and lower subjective well‐being was weaker for Asian Americans versus European Americans (Xu and Tsai 2023). However, Xu and Tsai also found that reflection was significantly associated with depression symptoms and poorer subjective well‐being to the same extent. In contrast, Niu (2013) found that brooding was significantly associated with depression symptoms for non‐Chinese adults, but not for Chinese adults and reflection was not associated with depression or suicidal ideation in either cultural group.

Studies examining other cultural groups yielded mixed results. Cheref et al. (2015) found that brooding and reflection were associated with depression symptoms and suicidal ideation in Black, Latino, and biracial groups, except reflection showed no association with suicidal ideation in the Latino group. One study found that while brooding and reflection were significantly associated with depression symptoms among racial/ethnic minority participants, among White individuals, only brooding but not reflection was significantly associated with depressive symptoms (Miranda et al. 2013).

### 3.6 Mediating and Moderating Variables

Some studies identified particular variables that may influence the associations outlined above. Schunk et al. (2021) found that for Germans, rumination was related to lower levels of life‐satisfaction through less social support, whereas for Japanese, social support did not mediate the relationship between rumination and lower life satisfaction. Choi and Miyamoto (2022) found that attributions of self‐doubt mediated the association between rumination and depression symptoms, partially explained the stronger association between rumination and depression symptoms in European Americans compared to Asian Americans. Tsai et al. (2011) found that happiness moderated the relationship between rumination and depression in European Americans, but not Asian Americans.

## 4 Discussion

This study synthesized existing research investigating cross‐cultural differences in the association between rumination and psychopathology outcomes. Overall, evidence of a moderating effect of culture on the association between rumination and psychopathology was mixed. Around half the studies (54%) included in the review focused on comparing members of Asian and Western cultural groups. Half of these studies found culture significantly moderated the association between rumination and psychopathology. Among these, a large majority found the association between rumination and psychopathology was weaker for those from Asian cultural groups compared to those from Western cultural groups, with only one study finding that rumination was more strongly linked to greater psychopathology in an Asian cultural group. In contrast, studies comparing different Western countries consistently found the associations between rumination and psychopathology were similar among these countries. The exploratory meta‐analyses highlighted a moderate positive association between rumination and depression symptoms. Yet there was no evidence to indicate that cultural group (Western; Asian) moderated the relationship between rumination and depression. However, this should be interpreted with caution given the few studies included and potential lack of statistical power to detect a cultural difference. Unfortunately, there were too few studies to conduct meta‐analyses for the other psychopathology outcome variables. These findings provide partial support for cross‐cultural differences in the association between rumination and greater psychopathology and importantly underscore the need for additional research to clarify the inconsistent findings.

Cultural theories predict weaker rumination effects on psychopathology among members of Eastern cultures. However, our meta‐analysis findings did not align with these theoretical predictions; there was no evidence of cultural group significantly moderating the association between rumination and depression symptoms. This discrepancy may stem from several factors. First, as noted above, even for depression symptomatology there were only a few studies (*n* = 9) included in the analyses and hence there was potentially a lack of statistical power to detect a cultural difference. Second, there was significant between‐study heterogeneity in terms of cultural grouping and measurement of symptoms. This may significantly impact the comparability of studies, while obscuring potential moderation effects. Third, it is possible that broad cultural categories (e.g., East/West, nationality/ethnicity) lack specificity to capture meaningful variation, while individual differences in cultural dimensions (e.g., individualism‐collectivism, interdependence, emotion regulation norms) might better explain variation in the effects of rumination on psychopathology. These dimensions may have varied significantly between studies, accounting for the non‐significant finding. Fourth, rumination may function differently in depression versus other conditions. While previous research has mostly focus on non‐clinical populations, it is possible that in the context of depression, the maladaptive nature of depressive rumination may override any cultural differences in rumination, creating more universal symptom patterns across cultures. Together, these considerations suggest that it is premature to discount the role of current cultural theories in understanding rumination in psychopathology and highlight that more nuanced approaches are needed in this area.

This review also identified three variables (social support, self‐doubt and happiness) that may help explain cultural differences in the relationship between rumination and psychopathology. Each of these variables were associated with rumination and psychopathology in ways that align with theoretical accounts of cultural differences in emotion regulation (e.g., de Vaus et al. 2018). First, Schunk et al. (2021) found for German participants, but not Japanese participants, social support mediated the relationship between rumination and lower levels of life‐satisfaction. The findings for the Japanese group suggest rumination may not erode social support as predicted by Response Styles Theory (Nolen‐Hoeksema and Davis 1999). This aligns existing cross‐cultural theories: for those from collectivistic cultures, rumination may function to support the interdependent self and maintain interpersonal harmony, through self‐reflection and self‐criticism (Kuppens et al. 2008). Additionally, individuals from Asian cultural groups tend to suppress negative emotions, which may protect against the erosion of social support associated with rumination (de Vaus et al. 2018; Nolen‐Hoeksema and Davis 1999). Second, Choi and Miyamoto (2022) found rumination was more strongly associated with depression symptoms among individuals who had a greater tendency to attribute rumination to self‐doubt (more common in Western participants). This finding is consistent with existing cross‐cultural theories integrating analytic thinking styles and independent self‐construals (de Vaus et al. 2018; Nisbett et al. 2010), wherein Western individuals are more likely to attribute distress to personal inadequacy, thereby amplifying rumination's maladaptive effects. In contrast, cultures with holistic thinking styles may engage more with contextual or situational factors, thus buffering the impact of rumination (Grossmann and Kross 2010). Third, Tsai et al. (2011) found happiness moderated the association between rumination and depression in European Americans, but not for Asian Americans. This finding was consistent with differences in emotion valuation that are proposed to affect rumination across cultures (de Vaus et al. 2018). Western cultures' emphasis on positive affect as a marker of wellbeing means rumination becomes more pathological when it contradicts expectations of happiness (de Vaus et al. 2018). In contrast, rumination may be less contingent on levels of happiness in Asian cultures, neither significantly buffered by it nor harmed by its absence, due to higher acceptance of affective fluctuations (Uchida and Kitayama 2009). Together, these three studies indicate the function of rumination may differ significantly between Western and Asian cultural groups and may be less maladaptive for those from Asian cultural groups in certain contexts. Their findings support existing frameworks explaining why cultural differences may emerge.

We also examined different subtypes of rumination and their association with psychopathology outcomes across cultures. Interestingly, all studies investigating subtypes of rumination found them to differ significantly in their associations with psychopathology across cultures. This supports conceptualisations of rumination as a multidimensional construct (e.g., Trapnell and Campbell 1999; Andrews and Thomson 2009) and highlights the importance of considering the form or process of rumination when considering outcomes. However, there were inconsistencies in the direction of these results. While two studies found brooding to be more strongly correlated with depression in a Western group and no cultural differences in the association between reflection and depression (Niu 2013; Xu and Tsai 2023), another study found reflection to be less associated with depression in the Western group and no cultural differences in the association between brooding and depression (Kwon et al. 2013). Xu and Tsai (2023) also found that problem‐solving analysis was less associated with depression and poorer mental health in Asians compared to Westerners, however this was the only study to examine this type of rumination. Thus, scarcity of relevant studies and their contrasting results limits any meaningful inferences about the directionality of associations between different forms of rumination and psychopathology. More research is needed to examine different forms of rumination, to clarify their role and influence on psychopathology cross‐culturally.

Only one study investigated cross‐cultural differences in the content of rumination. Tsai and Lau (2013) found that Asian and European Americans reported similar levels of distress when ruminating about an achievement failure, but the Asian group reported greater distress when reflecting on a negative interpersonal experience. This finding can be interpreted through understandings of cultural values; reflecting on social discord may be more distressing for cultural groups that prioritize upholding interpersonal harmony (de Vaus et al. 2018). However, as this study assessed distress immediately after rumination, it is unclear whether heightened distress persists over time. Immediate distress after rumination may not be maladaptive but rather needed for improvements in mood in the long term (Smyth 1998). Further research is needed to examine the content of rumination and outcomes of rumination longitudinally. Nevertheless, this finding suggests that the content of rumination may influence the outcome of rumination across cultures.

### 4.1 Methodological Considerations and Future Directions

This review identified several methodological limitations; limitations pertaining to (a) the measurement of rumination, (b) the measurement of culture, and (c) sampling issues. First, regarding the measurement of rumination, several studies utilized the RRS total as a measure of rumination. This may limit the meaningfulness of the results, given the brooding and reflection subscales represent different constructs, which may relate differently to psychopathology. Second, the RRS measures rumination in relation to depression (as expressed in the items). Despite depression being comorbid with many psychiatric conditions, the sole use of this instrument may not be appropriate when applied to other psychiatric disorders. For instance, Moulds et al. (2020) note theories of rumination in PTSD explicitly implicate trauma‐related rumination rather than general rumination in the maintenance of PTSD. Thus, the RRS may have less relevance in PTSD. In line with Moulds et al.'s suggestions, future research should include transdiagnostic and disorder‐specific measures of rumination (e.g., Repetitive Thinking Questionnaire, McEvoy et al. 2010; Perseverative Thinking Questionnaire, Ehring et al. 2011) when examining rumination in other psychological disorders. Furthermore, it is important that rumination measures are culturally appropriate and valid. Third, most research has focused on habitual rumination, by capturing participants' general tendency to ruminate. Only one study examined cultural differences in the content and themes of rumination. This is a key limitation given emerging research indicates the context and content of rumination influence psychopathology (Rector et al. 2008; Valdez and Lilly 2017). This is important to inform theoretical understandings and to better tailor treatments, particularly as applied in culturally and linguistically diverse groups. Although qualitative approaches examining the content of rumination have been utilized in previous rumination research (Birrer and Michael 2011; Newby et al. 2019; Newby and Moulds 2012), such methods have not extended to cross‐cultural research. Finally, the use of self‐report incurs potential bias. Individuals from collectivistic cultures tend to exhibit an acquiescent response style, characterized by a bias towards answering affirmatively to statements when completing self‐report questionnaires (Rammstedt et al. 2017).

The second set of limitations to emerge from the review relate to the measurement and classification of culture. Most studies used ethnicity or country as a proxy measure of an individual's cultural identity. However, there is significant within‐group variability of cultural norms, values, and processes among individuals from ethnic and racial groups (Markus and Kitayama 2010; Weiss et al. 2022). Thus, as the field develops, future research would benefit from further investigation of specific cultural factors (e.g., self‐construal, thinking style, values adherence) that may account for cross‐cultural differences in the relationship between rumination and psychopathology. Additionally, most cross‐cultural comparisons focused on Western versus Asian groups, with Asian groups limited to participants residing in large urban regions (e.g., China, Korea, Japan). Thus, there is a need for research to ensure representation of cultures that have not received attention (e.g., African, South American, Middle Eastern, First Nations), and rural contexts.

The third set of limitations pertain to sampling. Most studies examined healthy university students, with only three studies examining clinical populations. While such designs are an important first step in establishing potential cultural differences in rumination, it is essential future work investigates these relationships in clinical populations. Additionally, the review highlighted existing studies have focused disproportionately on depression symptoms (50%), highlighting the need to further research in less explored areas (e.g., PTSD, anxiety and eating disorders). The significant differences found transdiagnostically further emphasise the importance of investigating rumination other diagnostic areas.

### 4.2 Implications for Theory and Clinical Practice

This is the first systematic review to integrate cross‐cultural paradigms with clinical understandings of rumination in psychopathology. These findings contribute to the clinical literature by highlighting potential cultural differences in the association between rumination and psychopathology. Cross‐cultural theoretical accounts suggest that rumination, while generally considered a maladaptive process, may be associated with less negative psychological outcomes for individuals from certain cultural groups (i.e., Asians) under certain conditions, as rumination is congruent with cultural norms or values (e.g., de Vaus et al. 2018). However, the findings indicate the scarcity of sufficient studies to draw definitive conclusions about these theoretical propositions and highlight the need for further research in the area. There was some evidence that three culture‐specific mechanisms that may shape the rumination‐psychopathology relationship: (1) social support, which is less eroded by rumination in interdependent cultures, thereby buffering negative impacts on well‐being (2) self‐doubt attributions, which exacerbate distress in individualistic contexts through internalized causal analyses; and (3) happiness, which is associated with higher impact of rumination in cultures that emphasize positive affect (e.g., European Americans), as the dissonance between rumination and emotional ideals exacerbates distress. Despite this, it is important to note that at present psychopathological models of rumination (e.g., Nolen‐Hoeksema 1991; Foa et al. 2009; Williams et al. 2007) do not include culture as a variable. Thus, there is a clear need for theoretical accounts of rumination to integrate cross‐cultural psychology to better conceptualize rumination in non‐Western contexts. However, to inform such integration of models, it is imperative that further research is conducted to understand whether there are cultural differences and if there are differences, to clarify the variables and conditions driving potential cultural differences in rumination.

Our findings also raise some key clinical implications. The importance of cultural tailoring in clinical practice has been documented in longstanding clinical guidelines (e.g., American Psychological Association 2003; Australian Psychological Society 2006). Additionally, empirical evidence shows that treatment effects improve significantly when treatments are culturally tailored (Huey and Tilley 2018). Currently, clinicians need to be aware they are working with theoretical models of rumination that do not incorporate culture as a variable. This is a key issue given the centrality of rumination across various psychopathologies. Thus, clinicians need to consider culture (including values, worldviews, and self‐concept) as an important variable in assessment, formulation, and treatment. In practice, this may involve clinicians collaboratively determining with their clients whether treatment tasks targeting rumination are congruent with the individual's cultural norms and values. Importantly, this review highlighted further research is urgently required to provide more concrete guidelines for culturally tailored interventions targeting rumination.

### 4.3 Limitations of This Review

In addition to the limitations of the literature summarized above, we highlight some key caveats when interpreting the findings of this review. First, we did not review articles not written in English, which may have excluded some studies investigating non‐Western cultures. Second, although we explored cultural differences in Western versus Asian cultural groups, acculturation may be a confounding variable, given that some studies sampled Asians living in Western contexts (e.g., Asian Americans), while others sampled those living in their home country. Third, most of the studies reviewed utilized cross‐sectional designs, limiting causal inferences of findings. Additionally, given the preliminary nature of this study, we prioritized synthesizing studies comparing cultural groups and refrained from aggregating single‐country studies to infer cross‐cultural differences post hoc (e.g., Japan vs. Germany, as in Mezulis et al. 2004). While this approach avoids assumptions about cultural homogeneity within national samples and reduces risks of ecological fallacy or misclassification bias, it inherently narrows the scope of included evidence. Future research could expand on these findings by integrating single‐country studies, provided such analyses are grounded in robust theoretical frameworks to guide cultural classifications.

## 5 Conclusion

This systematic review highlighted some cultural differences in the association between rumination and psychopathology. While the exploratory meta‐analysis found no significant moderation by cultural group for depression symptoms, however this should be interpreted with caution given the few studies and lack of clinical samples employed in these studies. Therefore, the key findings from the review are highlighting the scarcity of sufficient studies to draw definitive conclusions about depression, the lack of robust evidence to support a clear cultural association between rumination and psychopathology, the need for further research examining the influences of culture on rumination in the context of psychopathology. Advancements in this research area is urgently needed to support the integration of culture into theories of rumination and enhance cultural sensitivity of clinical practise.

## Ethics Statement

As this study was a systematic review of published literature and did not involve direct contact with human participants or primary data collection, it did not require ethical approval from an institutional review board.

## Conflicts of Interest

The authors declare no conflicts of interest.

## Supporting information

Supplementary materials.

## Data Availability

The data that support the findings of this study are openly available in Open Science Framework at https://doi.org/10.17605/OSF.IO/RF6YS.
