# Supplementary material for: Cross‐Cultural Influences on the Association Between Rumination and Psychopathology: A Systematic Review
Source: J Clin Psychol. 2026 Mar 31;82(7):995–1015. doi: 10.1002/jclp.70135 (PMC13242247; doi:10.1002/jclp.70135)
Supplement: Supplementary file 1 — Supplementary materials. [file JCLP-82-995-s001.docx]

**Supplementary materials**Supplementary File 1: OVID Psycinfo Search Strategy

| **#** | **Query** |
| --- | --- |
| 1 | “Rumination (Cognitive Process)”/ |
| 2 | Ruminat*.mp. [mp=title, abstract, heading word, table of contents, key concepts, original title, tests & measures, mesh word] |
| 3 | (Repetitive and (thought* or thinking)).mp. [mp=title, abstract, heading word, table of contents, key concepts, original title, tests & measures, mesh word] |
| 4 | (Negative and (thought* or thinking)).mp. [mp=title, abstract, heading word, table of contents, key concepts, original title, tests & measures, mesh word] |
| 5 | (Perseverative and (thought* or thinking)).mp. [mp=title, abstract, heading word, table of contents, key concepts, original title, tests & measures, mesh word] |
| 6 | Brooding.mp. [mp=title, abstract, heading word, table of contents, key concepts, original title, tests & measures, mesh word] |
| 7 | Emotion regulation.mp. [mp=title, abstract, heading word, table of contents, key concepts, original title, tests & measures, mesh word] |
| 8 | Culture.mp. [mp=title, abstract, heading word, table of contents, key concepts, original title, tests & measures, mesh word] |
| 9 | Cultur* group*.mp. [mp=title, abstract, heading word, table of contents, key concepts, original title, tests & measures, mesh word] |
| 10 | Cross cultur* difference*.mp. [mp=title, abstract, heading word, table of contents, key concepts, original title, tests & measures, mesh word] |
| 11 | ((Culturally and linguistically diverse) or CALD).mp. [mp=title, abstract, heading word, table of contents, key concepts, original title, tests & measures, mesh word] |
| 12 | Socio cultural factor*.mp. [mp=title, abstract, heading word, table of contents, key concepts, original title, tests & measures, mesh word] |
| 13 | (Race or Racial).mp. [mp=title, abstract, heading word, table of contents, key concepts, original title, tests & measures, mesh word] |
| 14 | Ethnic*.mp. [mp=title, abstract, heading word, table of contents, key concepts, original title, tests & measures, mesh word] |
| 15 | Countr*.mp. [mp=title, abstract, heading word, table of contents, key concepts, original title, tests & measures, mesh word] |
| 16 | 8 or 9 or 10 or 11 or 12 or 13 or 14 or 15 |
| 17 | 1 or 2 or 3 or 4 or 5 or 6 or 7 |
| 18 | 16 and 17 |

**Supplementary file 2: Comprehensive Meta Analysis outputs**

**
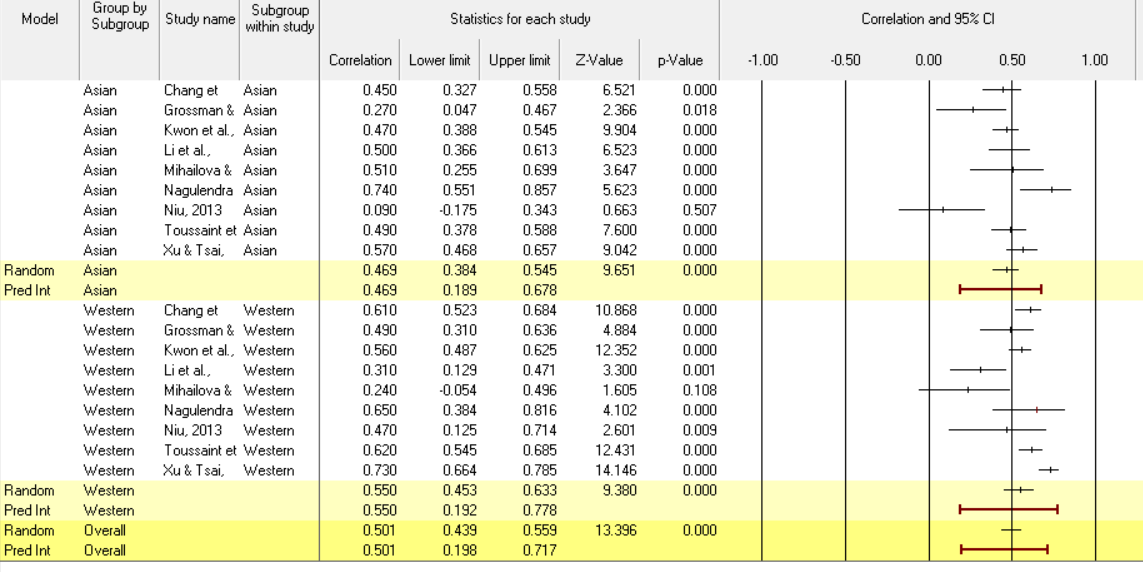
**

**
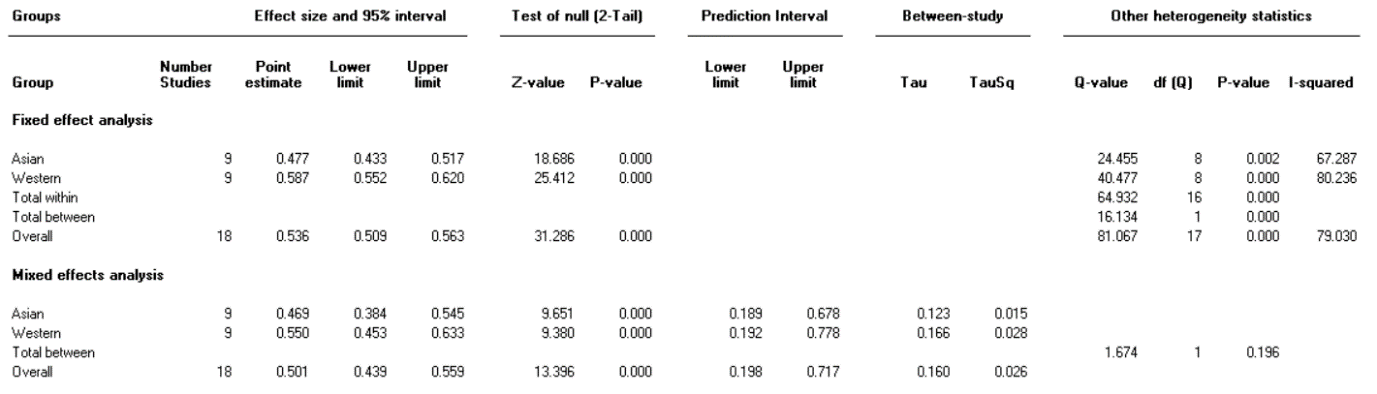

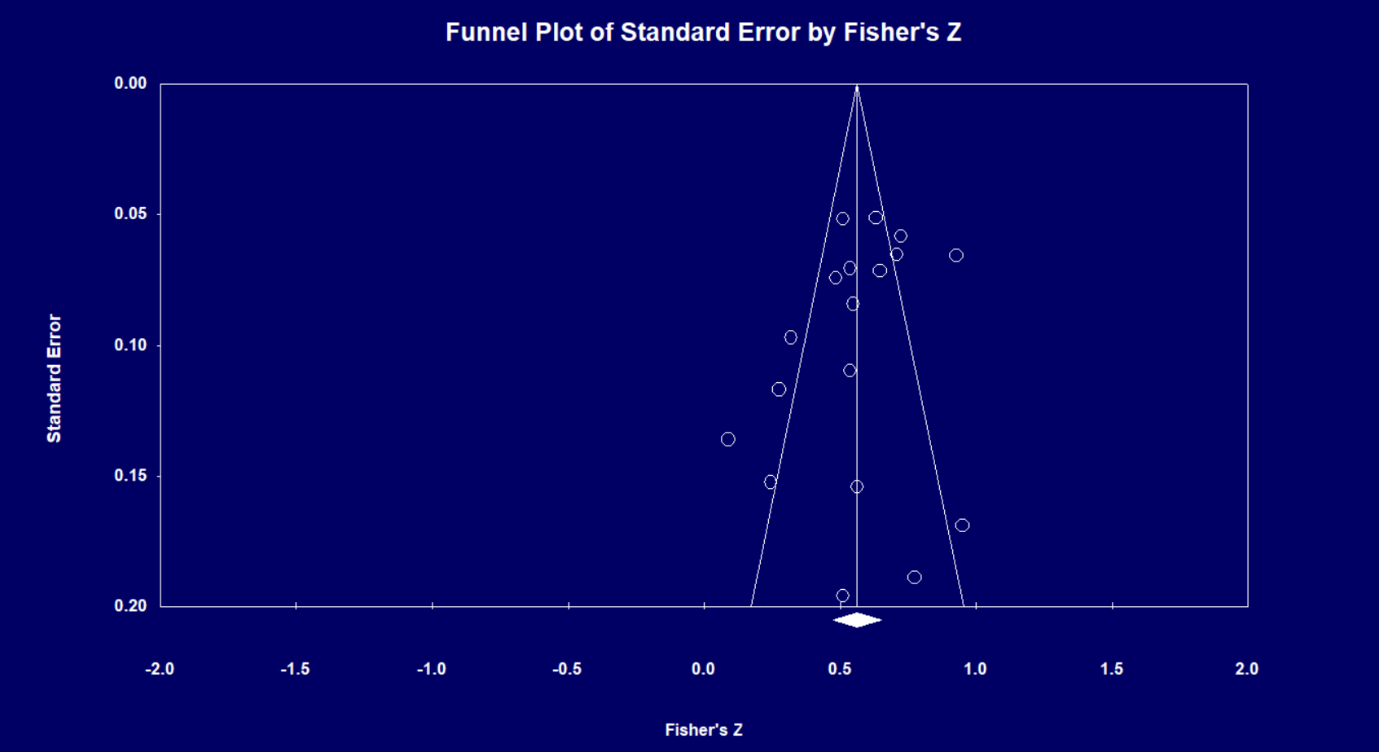

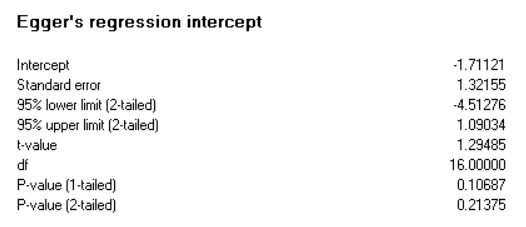

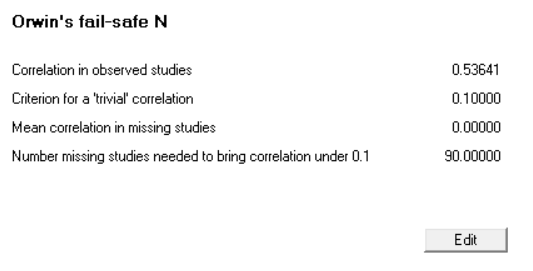
**

**Supplementary file 3**

*Ratings of study quality for included studies.*

|  | 01 | 02 | 03 | 04 | 05 | 06 | 07 | 08 | 09 | 10 | 11 | 12 | 13 | Total criterion met (out of 13) |  |
| --- | --- | --- | --- | --- | --- | --- | --- | --- | --- | --- | --- | --- | --- | --- | --- |
| Bravo et al., 2019 | Yes | Yes | Yes | No | Yes | Yes | Yes | No | No | No | Yes | Yes | Yes | 9 |  |
| Chang et al., 2010 | Yes | No | No | No | Yes | Yes | Yes | No | No | No | Yes | Yes | Yes | 7 |  |
| Cheref et al., 2015 | Yes | Yes | Yes | No | Yes | Yes | Yes | No | Yes | Yes | Yes | Yes | Yes | 11 |  |
| Choi and Miyamoto, 2022 | Yes | Yes | Yes | Yes | Yes | Yes | Yes | Yes | Yes | Yes | Yes | Yes | Yes | 13 |  |
| Eshun, 2000 | Yes | Yes | Yes | No | Yes | Yes | Yes | No | Yes | Yes | Yes | Yes | Yes | 11 |  |
| Grossman and Kross, 2010 | Yes | No | No | No | Yes | Yes | Yes | No | Yes | Yes | Yes | Yes | Yes | 9 |  |
| Jobson et al., 2022 | Yes | Yes | Yes | Yes | Yes | Yes | Yes | No | Yes | Yes | Yes | Yes | Yes | 12 |  |
| Kwon et al., 2013 | Yes | No | Yes | No | Yes | Yes | Yes | No | Yes | Yes | Yes | Yes | Yes | 10 |  |
| Li et al., 2022 | Yes | Yes | Yes | Yes | Yes | Yes | Yes | No | Yes | Yes | Yes | Yes | Yes | 12 |  |
| Mason and Lewis, 2017 | Yes | Yes | Yes | No | Yes | Yes | Yes | No | Yes | Yes | Yes | Yes | Yes | 11 |  |
| Mihailova and Jobson, 2020 | Yes | Yes | No | Yes | Yes | Yes | Yes | Yes | Yes | Yes | Yes | Yes | Yes | 12 |  |
| Miranda et al., 2013 | Yes | No | Yes | Yes | Yes | Yes | Yes | No | No | Yes | No | Yes | Yes | 9 |  |
| Nagulendran and Jobson, 2020 | Yes | Yes | No | Yes | Yes | Yes | Yes | No | Yes | Yes | Yes | Yes | Yes | 11 |  |
| Niu, 2012 | Yes | Yes | Yes | Yes | Yes | Yes | Yes | No | Yes | Yes | Yes | Yes | Yes | 12 |  |
| Potthoff et al., 2016 | Yes | No | No | No | Yes | Yes | Yes | No | Yes | Yes | Yes | Yes | Yes | 9 |  |
| Ramos-cejudo et al., 2017 | Yes | Yes | Yes | No | Yes | Yes | Yes | No | No | No | Yes | Yes | Yes | 9 |  |
| Schunk et al., 2021 | Yes | Yes | Yes | Yes | Yes | Yes | Yes | Yes | Yes | Yes | Yes | Yes | Yes | 13 |  |
| Schunk et al., 2022 | Yes | Yes | Yes | Yes | Yes | Yes | Yes | Yes | Yes | Yes | Yes | Yes | Yes | 13 |  |
| Schunk et al., 2023 | Yes | Yes | Yes | Yes | Yes | Yes | Yes | Yes | Yes | Yes | Yes | Yes | Yes | 13 |  |
| Taku et al., 2009 | Yes | No | No | No | No | Yes | Yes | No | No | No | Yes | Yes | Yes | 6 |  |
| Toussaint et al., 2021 | Yes | Yes | Yes | Yes | No | Yes | Yes | No | Yes | Yes | Yes | Yes | Yes | 11 |  |
| Tsai et al., 2011 | Yes | Yes | No | No | Yes | Yes | Yes | No | No | No | Yes | Yes | Yes | 8 |  |
| Tsai and Lau, 2013 | Yes | No | Yes | Yes | Yes | Yes | Yes | No | Yes | Yes | Yes | Yes | Yes | 11 |  |
| Xu & Tsai, 2023 | Yes | Yes | Yes | Yes | Yes | Yes | Yes | No | Yes | Yes | Yes | Yes | Yes | 12 |  |
| *Note.* 01 = Were the research questions clearly outlined? 02 = Was the population clearly described and representative? 03 = Did the study justify sample size? 04 = Were the criteria for inclusion and exclusion in the sample clearly defined? 05 = Were the study participants and the setting described in detail? 06 = Was rumination measured in a valid and reliable way? 07 = Was an appropriate standard used to measure cultural group? 08 = Did the study identify missing data and how was it dealt with? 09 = Were confounding factors identified? 10 = Were strategies to deal with confounding factors stated? 11 = Was wellbeing/psychopathology measured in a valid and reliable way? 12 = was appropriate statistical analysis used? 13 = Were limitations reported? | | | | | | | | | | | | | | | |
